# In‐Situ Measurements of Radiofrequency Electromagnetic Fields Measurements Around 5G Macro Base Stations in the UK

**DOI:** 10.1002/bem.70012

**Published:** 2025-06-30

**Authors:** Carolina Calderon, Darren Addison, Azadeh Peyman

**Affiliations:** ^1^ UK Health Security Agency (UKHSA) Chilton Oxfordshire UK

**Keywords:** exposure assessment, public exposure, telecommunications

## Abstract

Radiofrequency (RF) electromagnetic field spot measurements were performed in line‐of‐sight to 56 active 5G macro base stations across 30 publicly accessible locations in the United Kingdom (UK). Four different exposure scenarios were assessed: background (no traffic instigation), streaming videos, downlink speed test, and extrapolation of SS‐RSRP decoder measurements. Power density measurements across the 420 MHz–6 GHz frequency range were also performed at each site to assess the total exposure from various RF sources in the environment. Both total RF and 5G specific power density levels were found to be well within the 1998 ICNIRP public reference levels, even when extrapolating to worst‐case scenario (≤ 5%). 4G downlink was the dominant contributor to total RF exposure, with 5G contributing on average less than 10%. No statistically significant difference was observed between beamforming and non‐beamforming sites. Streaming did not seem to contribute materially to exposure levels, suggesting that background measurements are a good representation of typical downlink exposure at current urban and suburban 5G sites.

## Summary

- Power density spot measurements close to 56 5G macro base stations in the UK were performed following IEC 62232 62232:2025.
- Extrapolation to maximum exposure was ≤ 5% of the ICNIRP 1998 public reference levels across all base stations.
- No statistically significant difference was observed between beamforming and non‐beamforming sites.

**Abbreviations**
ARFCN: absolute radio‐frequency channel number
PCI: physical cell identity
RBW: resolution bandwidth
SSB: synchronization signal/PBCH
SS‐RSRP: synchronization signal reference signal received power
VBW: video bandwidth

## 1 Introduction

Ever‐increasing demands on connectivity, data throughput, and energy efficiency have fueled the perpetual development of communication systems and associated infrastructure. In 2020, a UK government initiative was put in place to ensure that 95% of the UK would have 4G coverage by the end of 2025 (DCMS 2020), and a similar initiative was also put in place to support 5G deployment (DSIT 2023). These initiatives resulted in the upgrading of thousands of sites with a combination of additional spectrum and higher operating power, as well as the installation of 190 new base stations since 2020 (Ofcom 2023). In parallel, 2G and 3G systems are also being phased out and replaced by 4G and 5G systems, with plans of completely phasing out 3G in 2025 (Ofcom 2024a).

The UK mobile telecommunications industry has voluntarily committed to ensuring their radiofrequency (RF) transmitting equipment comply with the international electromagnetic fields (EMF) exposure guidelines (DSIT/DCMS 2022). However, new developments always raise concerns amongst members of the public. This has been particularly the case during the introduction of the 5G New Radio (NR) system, hereafter referred as 5G. Some of the concerns arise from the fact that 5G uses Massive Multiple Input Multiple Output (MaMIMO) technology, whereby the base station (BS) uses beamforming to optimize communication with the user equipment (UE), such as a mobile phone. This communication system may also soon use higher frequencies than those used by current mobile phones (Frequency Range 2: 24.25–71.0 GHz) and will require further BS densification. All this novelty has been met with increased public apprehension.

The UK Health Security Agency (UKHSA) advises the UK Government on the public health aspects of exposure to EMFs, including those from mobile phone base stations and other radio transmitters in the environment.

In Europe, there have been several studies looking into the EMF exposure around 5G base stations (Aerts et al. 2021, Christopoulou 2024, Deprez 2022, Onishi 2023, Selmaoui 2021) while in the UK, the only publicly available data currently comes from audits performed by the Office of Communications (Ofcom), the mobile telecommunication regulator (Ofcom 2020; Ofcom 2024b). These audits are overall assessments of the radio spectrum without focus on 5G. Because of the beamforming capabilities of 5G, these assessments do not consider exposure to a person using 5G on their phone. Instead, they provide snapshots of the background exposure from all RF sources present in the environment.

To compliment Ofcom data with specific focus on assessing RF exposure from 5G in the UK, UKHSA set out to perform measurements in publicly accessible areas near active 5G BSs (FR1 frequency band: 410–7125 MHz), with the aim to fill this knowledge gap, and compare 5G exposure to other environmental sources of RF, as well as comparing exposure between beamforming and non‐beamforming 5G sites. Exposure was also assessed for various traffic loads, using various exposure assessment techniques stipulated in the IEC 62232 standard (2025), across a number of urban and suburban sites. This study also aimed to demonstrate the applicability of the IEC 62232 standard methods when performing in‐situ measurements on active 5G sites, with limited network information.

## 2 Materials and Methods

### 2.1 Base Station Sites

**Figure 1:**
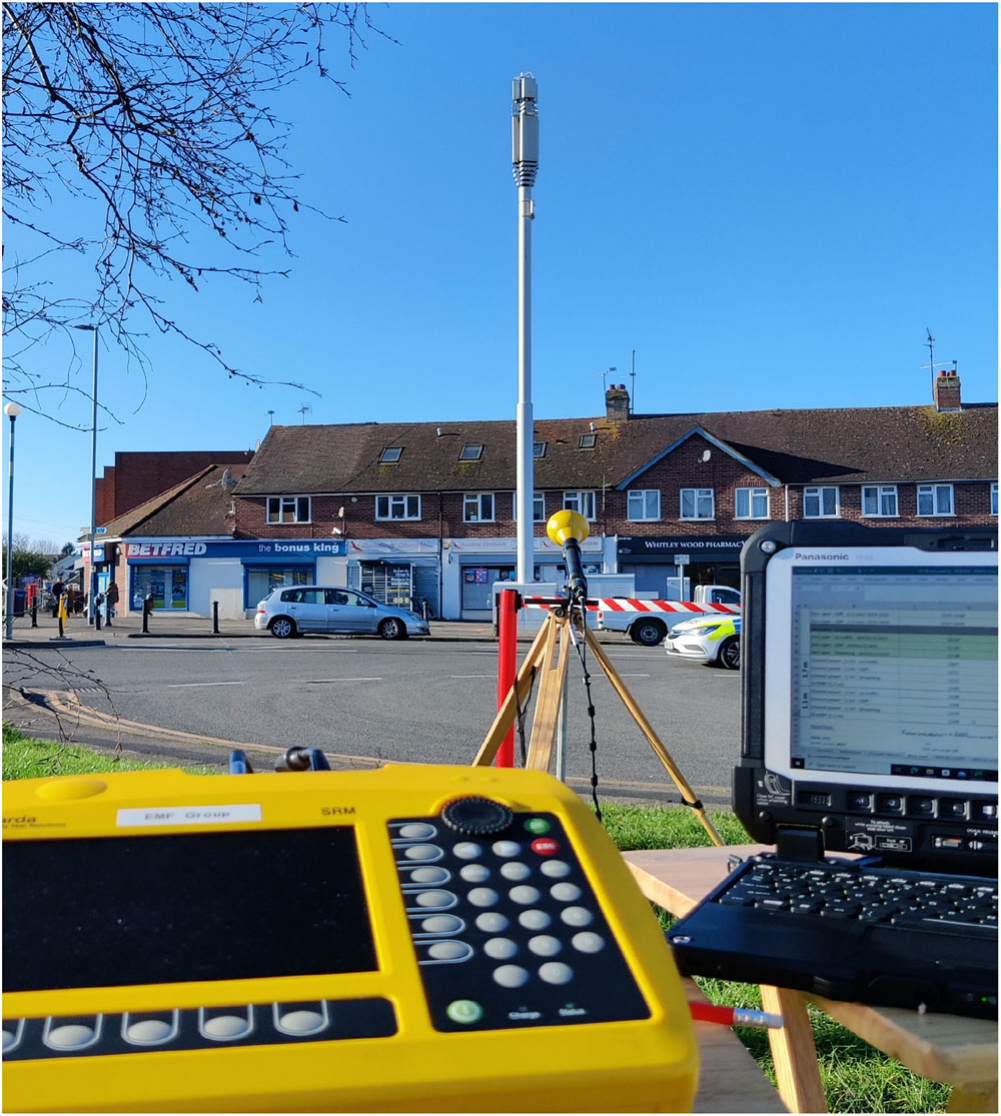
Example of the measurement set up.

A convenience sample of sites were selected, based on the availability to perform measurements in publicly accessible areas and with line‐of‐sight (LOS) to one or several 5G BSs (Figure 1), sampling a variety of microenvironments (commercial, industrial, residential, parks), BS types (Roof‐top, Lattice structure, StreetWorks), Mobile Network Operators (MNOs), and geographic locations within southern England (London, Swindon, Reading, Oxford, Bristol, Abingdon and Newbury). BSs employing beamforming as well as those with fixed beams were also sampled for comparison. All sites investigated were Non‐Stand‐Alone (NSA), meaning the core network was provided through 4G. MNOs were contacted to confirm 5G BS location, and obtain parameters such as frequency bands, SS‐RSRP synchronization signal block (SSB) broadcast frequency, and whether power of the BS was capped (sometimes referred as power lock). They were also asked whether site sharing or Dynamic Spectrum Sharing (DSS) were used at any given site, and whether multiple 5G carriers were being used. Permission was sought from local councils or from site owners before measurements.

### 2.2 Measurement Protocol

The protocol for measurements was based on the IEC 62232 standard (2025), using a Narda Selective Radiation Meter 3006 (Narda Safety Test Solutions [STS], Pfullingen, Germany), hereafter referred as SRM, connected to a three‐axis isotropic electric field sensor, model 3502/1 with a frequency range of 420 MHz–6 GHz (Narda STS). The sensor was mounted on a wooden tripod and connected to the SRM via a calibrated Narda RF 5m 3602/02 cable (9 kHz–6 GHz, [Narda STS]), minimizing the influence of body presence during measurements. The expanded measurement uncertainty (of the sensor, the cable and the SRM basic unit) was +2.6 /−3.8 dB or better, depending on the frequency, as specified by the manufacturer (Narda STS). The equipment was under manufacturer calibration.

In the case of 5G BSs using beamforming, downlink (DL) traffic is sent through beams which are narrower than the 120° sector beams typically used in previous communication systems. Thus, to ensure that the measurement equipment captures the 5G signal, DL traffic needs to be instigated by a user equipment (UE), such as a mobile phone. The measurement equipment should then be placed in the beam directed to the UE. To achieve this, a 5G enabled mobile phone was used with different SIM cards related to each of the MNOs, and an unlimited 5G data plan which prioritized fastest speeds.

### 2.3 Traffic Generating

Two mobile phone applications were used: a speed test application (nPerf, version v2.14.0), to generate DL traffic, and a network optimization application (Cellular‐Pro, Version V.1.6.2) which provided network parameters such as PCI, ARFCN, SSB frequency, bandwidth, and center frequency. When the UE was used to generate traffic, it was placed at least 2 m behind the measurement equipment but aligned with the BS and the measurement point. The UE was otherwise set to flight mode during measurements.

### 2.4 Measurement Scenarios

Three evaluation scenarios were considered, as following:

*Snapshot of all environmental RF sources:* These were 6‐min average power density measurements of all RF sources in the frequency range of 420 MHz–6 GHz, using the Spectrum Analyser Mode of the SRM. The power density was integrated over various RF bands (Supporting Information S1), excluding any measurements below noise floor (< 1 µW/m^2^). Background measurements (taken in an anechoic chamber with same settings) were subtracted from the data.

*Channel power measurement of the 5G signal*: To get an estimation of the 5G RF exposure from the BS when a person is using their phone (not uplink), channel power measurements (Spectrum Analyser mode with integration over the 5G band, RBW = 1 MHz, VBW = OFF, Trace = Average) of the 5G signal under investigation were performed under the following scenarios: no traffic generated (background), DL streaming test, and DL speed test. Due to limitations on the nPerf app settings, the measurement averaging time for the streaming and speed tests were limited to 1 min, as opposed to 6 min (used for background measurements). The speed test app would actively specify which MNO and technology (4G/5G) was being used during tests, and this was monitored to ensure traffic was captured from 5G. Background measurements were not taken for these measurements; thus, the equipment noise would still be present within the measurement results, which may lead to a small overestimation of exposure.

*Extrapolation to worst‐case exposure*: Based on the IEC 62232 (2025) standard, the broadcast 5G signal was measured using the dedicated 5G decoder mode of the SRM, which measures the average power density per resource element (RE) of the SSB bursts, $S_{\mathrm{SSB}}$, for a given SSB frequency. The results are provided per beam, and the beam with the highest power density was used for the calculations. This measurement was then scaled to: the whole bandwidth of the 5G band under investigation ($F_{\mathrm{BW}}$, using Table E.11 from IEC 62232 (2025) and for the relevant subcarrier spacing, the maximum DL duty factor ($F_{\mathrm{TDC}}=0.75$), and any beam pattern or transmission power (Tx) differences between traffic and broadcast signal ($F_{\mathrm{SSB}2\mathrm{Traffic}})$:

$$S_{\mathrm{extrapolated}}=\;S_{\mathrm{SSB}}\cdot F_{\mathrm{SSB}2\mathrm{Traffic}}\cdot F_{\mathrm{BW}}\cdot F_{\mathrm{TDC}}$$

where $F_{\mathrm{SSB}2\mathrm{Traffic}}=\frac{S_{\mathrm{traffic},\mathrm{scope}}}{S_{\mathrm{SSB},\mathrm{scope}}}$ is the ratio between the traffic signal, $S_{\mathrm{traffic},\mathrm{scope}}$, and the broadcast signal, $S_{\mathrm{SSB},\mathrm{scope}}$, both of which were determined through 40 ms scope measurements at the SSB frequency during DL speed tests (RBW = 1 MHz, VBW = OFF, time resolution of 36 µs, Output = “Avg”‐ equivalent to RMS detector). The first 20 ms seconds of data were overplotted over the last 20 ms of data from each trace to identify the SSB burst, and $S_{\mathrm{traffic},\mathrm{ZS}}$ was taken as the maximum of the traffic samples. The SSB frequency and the subcarrier spacing of the base station were obtained through the optimization network app (nPerf).

Measurements were performed for each of the three orthogonal axes of the probe sequentially, to ensure the necessary time resolution allowing observation of the SSB bursts. Performing a scope measurement at a frequency away from the broadcast signal but within the 5G band in question would provide a similar result to estimating $S_{\mathrm{SSB}}\cdot F_{\mathrm{SSB}2\mathrm{Traffic}}$. However, for the purpose of this study, it was deemed best to also measure the broadcast signal with a decoder, which allowed confirming that the measured signal was from the BS being assessed (using PCI). This also allowed checking consistency between scope and decoder measurements.

$F_{\mathrm{SSB}2\mathrm{Traffic}}$ is equal to the beam extrapolation factor in IEC62232 (2025) for beamforming antennas, and equal to the inverse of the boosting factor for non‐beamforming antennas. It was not possible to get information from all operators regarding whether base stations were actively limiting the time‐averaged transmitted power to avoid exceeding a configured threshold (power lock function), thus the power reduction factor used in IEC62232 was set to 1 as a conservative measure.

Channel power and $S_{\mathrm{SSB}}$ measurements were performed at three heights (1.1, 1.5, 1.7 m) and averaged together. Results were compared with the guidelines set out by International Commission on Nonionizing Radiation (ICNIRP), by dividing the measured power densities by the power density reference levels for the general public (ICNIRP 1998), a quantity referred to as exposure ratio (ER). The ICNIRP 1998 were used as the current UK government policy on public exposure refers to these guidelines, following the adoption of the 1999/519/EC EU council recommendation. It should be noted that, for the frequency range investigated here (420 MHz–6 GHz), the reference levels for power density (whole body) are the same for both ICNIRP 1998 and ICNIRP 2020. The only difference is that the former applies an averaging time of 6 min as opposed to 30 min. Given the number of measurements being performed at each site (for the various exposure scenarios, methods, and for the various operators), a 30‐min averaging time would not have been feasible. Variations in 5G traffic at any given time may affect the repeatability of the background and streaming test measurements when comparing the two different averaging times.

To monitor the level of traffic during measurements, parallel zero span measurements were performed during channel power measurements, at the center frequency of the 5G band investigated and with maximum RBW (40 MHz), using a directional Log periodic antenna (Aaronia_HyperLOG_60100) connected to an Anritsu Field Master Pro MS2090A Spectrum Analyser via a 5 m RG400 cable, placed at least 1.5 m away from the SRM sensor. The sweep time was set such that each time step was equal to 1 OFDM symbol and that at least two frames were sampled (> 20 ms). Measurements with this equipment are not reported here, as the main purpose of these measurements was to monitor traffic during channel power measurements.

All measurements were made during the working day, and during the period of March 2023 and May 2024.

All statistical analyses were made using Microsoft Excel 365 and R.

## 3 Results

Site sharing was commonly observed, resulting in a total of 56 LOS BSs assessed across the 30 sites surveyed, 21 of which used beamforming. Mean distance to BS was 94 m (21–360 m). The sample contained a balanced amount of BS types (lattice *N* = 15, rooftop *N* = 14, StreetWorks *N* = 27). BSs from all UK's main MNOs were successfully sampled (*N* = 10, 12, 13 and 21).

In terms of microenvironment, 18 BSs were in a commercial environment, 17 in a residential area, 13 in an industrial one and 4 were located inside a park. The remaining BSs were in a college and a leisure center and were classified as “other.” All but 3 of the sites used Frequency Division Duplex (FDD) instead of time division duplex (TDD).

Average extrapolated exposure across all BSs was 0.6% of the ICNIRP 1998 public reference levels, with a range of 0.0005%–5%. The combined extrapolated exposure of all LOS BSs at any given site was only marginally higher, with an average of 1% across all sites and a range of 0.1%–6%. The average power density for the various exposure scenarios assessed are shown in Table 1.

**Table 1:** Power densities for the various exposure scenarios assessed.

Scenario	Arithmetic mean, mW/m^2^	Geometric mean, mW/m^2^	Minimum, mW/m^2^	Maximum, mW/m^2^
Background	0.5	0.2	0.001	4
Streaming test	0.7	0.2	0.001	6
Speed test	15	4	0.01	122
Extrapolation	64	20	0.02	457

**Figure 2:**
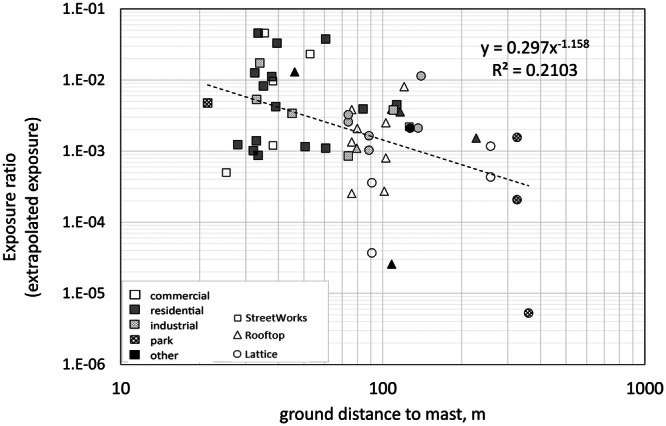
Exposure ratio (from extrapolation) as a function of ground distance to BS.

Regression analysis showed a significant (*p* < 0.001) trend between 5G EMF power densities and distance to BS, but a power function fitted poorly to the data (*r* ^2^ = 0.08), with exposures typically varying by several orders of magnitude at any given distance (Figure 2). Including other potentially exposure‐relevant parameters in the regression, such as beamforming, BS type and microenvironment, did not yield any significant dependence. Similar results were also observed when analyzing SS‐RSRP measurements as opposed to the extrapolated exposure ratio.

**Figure 3:**
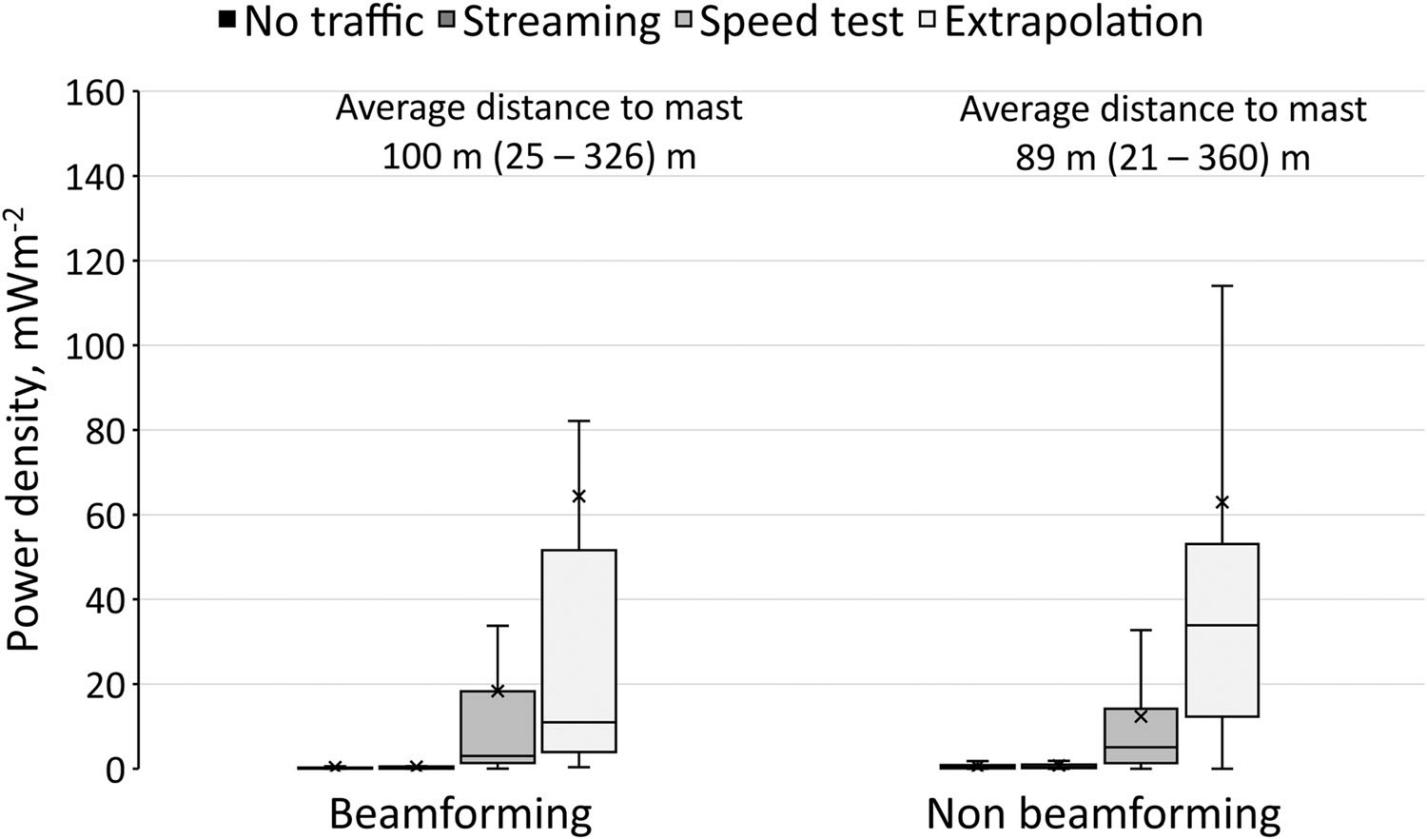
Channel power density for beamforming (*N *= 21) and non‐beamforming (*N *= 35) sites, and for the various assessment scenarios. The upper and lower edges of the boxes represent the 75th and 25th percentile respectively, while the median is shown as a horizontal line across the box, and the mean as a cross. The lower limit of the whiskers shows the minimum, while the upper limit of the whiskers shows the maximum, excluding outliers which were over 1.5 times the interquartile range.

For all four types of assessments performed, exposure from 5G at beamforming sites did not seem to be significantly different from non‐beamforming sites (Figure 3). When considering how much higher channel power measurements were during speed tests compared to background measurements for any given BS, the results were not statistically different for beamforming sites (*p* = 0.4), with an average ratio of 82 (4–502) versus 57 (1–582) for non‐beamforming sites.

**Figure 4:**
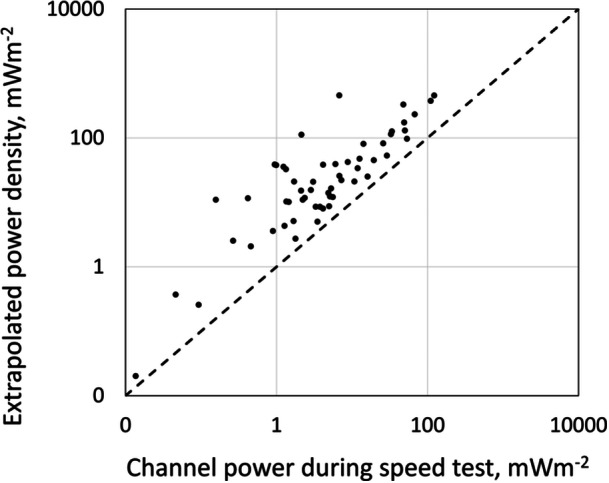
Channel power measurements during speed tests vs extrapolation of the SS‐RSRP measurements. The dotted line is the line of equality, and shows that the extrapolated power density is always greater than the channel power during downlink speed tests.

Power densities during streaming tests were found to be similar to background levels (Figure 3). Power densities during speed tests, on the other hand, were found to be lower or equal to the extrapolation of the SS‐RSRP decoder measurements (Figures 3 and 4).

**Figure 5:**
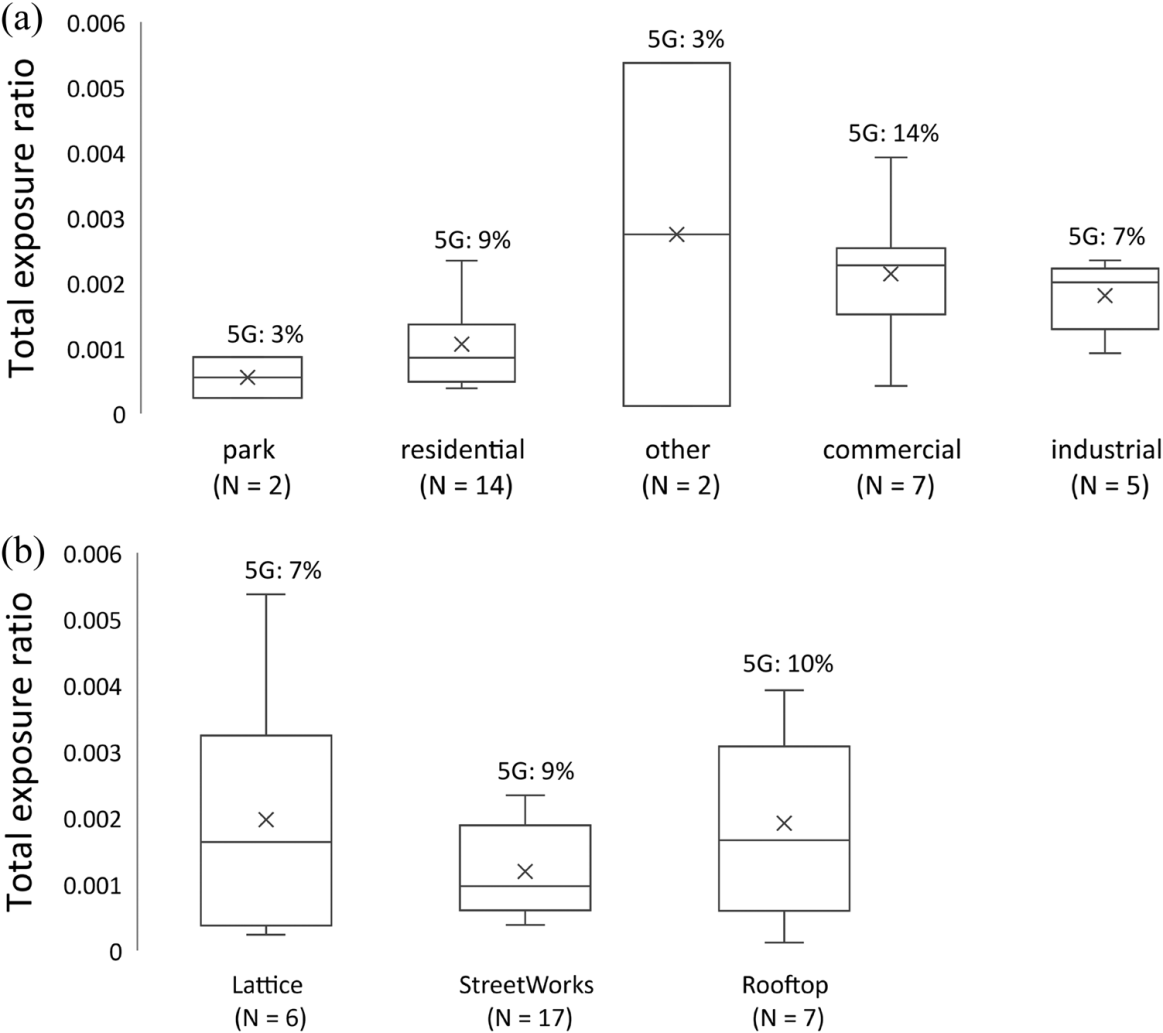
Total RF exposure across the various microenvironments (a), and across various BS types (b). The percentage of the 5G contribution to total exposure is shown above each box.

Total exposure from RF sources across the 420 MHz–6 GHz frequency range were below 1% of the 1998 ICNIRP public reference levels, with a mean of 0.2% and a range of 0.01% – 0.5%. Type of microenvironment and BS type did not show to have an impact on the total RF exposure (Figure 5). Average contribution of 5G to total RF exposure was 9% (range: 0.6%– 38%) and was not found to be significantly dependent on microenvironment nor BS type.

5G was the most dominant source in only 2 of the 30 sites (1 residential site and 1 commercial site), while 4G DL was the dominant source in 25 sites (83%). Of the sites where 5G was not the dominant background source, exposure from the dominant source was found to be less than the total extrapolated 5G exposure (at that site) in 84% of the sites (21 out of 25).

Summing all 5G background channel power measurements at a given site was found to be similar to the 5G exposure obtained from environmental measurements; the ratio of the total 5G environmental measurements and the sum of the channel power background measurements had an average of 1.25, with a range of 0.3–6.1 across sites. The environmental measurements captured all 5G signals at the site, even NLOS.

For the decoder measurements, variation across the three heights investigated varied on average by 70% (4%–160%); the median relative standard deviation between the three values at the 3 heights was 32% overall (average: 36%, range: 2%– 83%). No difference was observed between beamforming (median: 27%, average: 30%) and non‐beamforming (median: 37%, average: 39%) sites. When obtaining the SS‐RSRP value from the scope measurements as a consistency check with the decoder measurements, the median difference between the two estimates at the same height (1.5 m) was 21% (average: 31%), and the Pearson correlation between the average from the three heights and the value obtained from scope measurements was 0.88.

## 4 Discussion

The deployment of 5G together with the overall densification of the mobile network has generated public concerns that RF exposure is increasing. Ofcom BS audits from 2001 to 2007 yielded median exposure levels five orders of magnitude lower than exposure guidelines, across 1809 sites (Mann 2011). In this study, the median exposure level across the whole RF spectrum investigated was three orders of magnitude lower than exposure guidelines. Ofcom has not reported median levels of their more recent audits, but in their 2020 summary (Ofcom 2020) they reported a maximum of 1.5% of the public reference levels which is similar to what is reported here. This could suggest that RF exposure levels in the environment are higher than 20 years ago, albeit still several orders of magnitude below exposure guidelines. It should be emphasized, however, that mean distances to base stations is not reported by Ofcom, and it is unclear whether measurements were made in LOS. Both aspects limit the comparison between Ofcom and UKHSA measurements.

In France, a small increase in total EMF exposure was observed 8 months after 5G deployment (Sefsouf et al. 2024), but authors believed it to be due to the completion of 4G deployment rather than 5G. In this study, 4G DL was the dominant contributor to exposure, and contribution from 5G to total background exposure was found to be only 8.9% on average (range: 0.6%–37.6%). This is in line with studies in other countries, with Greece reporting a contribution of less than 6% (Christopoulou et al. 2024) and South Korea reporting a contribution of 15% of total telecommunication emissions (Selmaoui et al. 2021). Switzerland reported a contribution of less than 1% during background measurements (Aerts et al. 2021), while in Japan 5G emissions were found to be 2 to 3 orders of magnitude lower than total RF emissions (Onishi et al. 2023). Most of the measurements in literature were performed during the period of 2019 and 2022, when 5G started to emerge and thus low background 5G activity would have been expected. In the UK, Ofcom reported that the proportion of mobile connections on 5G was still only around 20% in 2024 (Ofcom 2024c). This is over the whole network and reflects the proportion of 5G BSs compared to 4G BSs as well as the proportion of 5G data plan subscriptions; it does not provide the proportion of 5G connections in areas of 5G coverage. Nevertheless, 5G contribution to total RF exposure is likely to increase as 5G subscriptions become more prevalent.

One might wonder whether the increase in DL traffic over the years could have also potentially increased exposure levels. However, mobile networks have become more efficient as technology evolved. In the UK, the monthly average data per user has increase from 500 MB (Megabytes) in 2013, to 5.3 GB (Gigabytes) in 2021 (Ofcom 2022), while typical DL speeds are around seven times (4G) to 66 times (5G) faster than 3G, respectively (U‐switch 2023). The fast DL speeds in 5G might explain why exposure during streaming tests was found to be similar to background levels; A High‐Definition video takes 3 GB/hour of data (~7 Mbps) and a high‐quality audio uses 144 MB/hour of data, while 5G allows for 134 Mbps on average (U‐switch 2023), meaning it doesn't require much DL time to stream a video. Given that streaming videos is the most data intensive activity most people are likely to undertake, the results presented here suggest that making RF environmental measurements is sufficient to characterize typical DL exposure to the public in urban environments, irrespective of whether they are using a mobile phone or not. Background 5G exposure levels, i.e. without DL traffic instigation, were found to be similar to those reported in other countries, with mean background 5G exposure levels of 0.07 mWm^−2^ (max = 3.95 mWm^−2^), 0.08 mWm^−2^ (max = 44.37 mWm^−2^), and 0.14 mWm^−2^ reported in France, South Korea and Greece ([Sefsouf et al. 2024; Selmaoui et al. 2021; Christopoulou et al. 2024], Supplement 2). In Japan, car‐mounted measurements yielded levels of a couple of µWm^−2^ ([Onishi et al. 2023], Supporting Information S2) across the 5G band, and lower levels were reported in Switzerland, possibly due to the due to the restrictive EMF limits applicable in Switzerland resulting in lower base station powers (Aerts et al. 2021).

Measurements during speed tests (Table 1: 15 mWm^−2^, (0.01–122) mWm^−2^) were found to be similar to those reported by Deprez et al. who reported an average of 18 mWm^−2^ (0.7 – 59) mWm^−2^ at distances between (62–300) m across six base stations ([Deprez et al. 2022], Supporting Information S2). Lower measurements were reported in South Korea ([Selmaoui et al. 2021], Supporting Information S2), where 0.22 mWm^−2^ were measured at 150 m from a single 5G base station with maximum download traffic and a 20 MHz channel bandwidth. Sefsouf et al. 2024 did not measure at maximum download traffic, but instead their protocol consisted of measurements while downloading a single 1 GB file over 6 min which the authors estimate to equate to around 4% duty factor. This resulted in channel power measurements of up to 87.70 mWm^−2^ (average: 1.30 mWm^−2^) across 464 sites, in line‐of‐sight and around 100 m from the base stations. Again, lower levels were reported in Switzerland ([Aerts et al. 2021], Supporting Information S2).

Extrapolation to worst‐case exposure was found to be well within the public ICNIRP 1998 reference levels. This level of exposure is, however, unlikely. Channel power measurements during speed tests were on average around 28% of the extrapolated exposure levels, for both beam‐forming and non‐beamforming sites. This is partly because a duty factor of 75% was not generally feasible, and in some cases, it was far lower. It was expected that this would be mainly due to the presence of other users in the network and would be more pronounced for beam forming sites, but the difference between extrapolated exposure and channel power during speed tests was the same for both types of BSs. In fact, there was no statistically significant difference in exposure between beamforming and non‐beamforming sites.

Given the various exposure‐relevant parameters that are site dependent, it was expected that distance on its own would not be a very good predictor of exposure, even when just considering the 5G broadcast signal ($S_{\mathrm{SSB}}$) i.e. excluding variations in channel bandwidth and traffic loads. Differences in output power and mast location (e.g. rooftop vs*.* Streetworks) as well as layout of reflectors will have an impact on the EMF levels. Adding some of the exposure relevant parameters into the regression did not yield more significant difference. Thus, although exposure was found to decrease with distance to BS, this study did not find a way to predict the levels, based on the site parameters obtained.

Several studies have reported higher exposures in dense urban or industrial areas compared to rural areas (Selmaoui et al. 2021; Loizeau et al. 2023; Iakovidis et al. 2022). This study did not sample rural sites, only microenvironments in urban/suburban areas, where 5G deployment was prioritized. Measurements were specifically made close to base stations and therefore the measurements presented here were inherently biased to locations of higher exposure and thus results are not necessarily representative of the microenvironment they were in. Nevertheless, the purpose of this assessment was to sample a variety of urban/suburban environments; and results suggest that EMF exposure level close to base stations did not seem to depend on the type of environment.

The main limitation of this study was the lack of control over the network. This had several implications. First, during channel power measurements, the UE would either revert to 4G, which was evident from what was reported by the network app (nPerf) or, occasionally, would change to a different 5G frequency band. The parallel zero span measurements with the directional antenna helped check that the 5G band being evaluated was being used by the UE during measurements. This was mainly informative for the speed tests, as it wasn't always evident during streaming tests due to background traffic. When it was evident that the 5G band in question was not being used, the measured data were excluded from the analysis. Making a simple spectral measurement over the whole RF spectrum while instigating traffic may circumvent the problem of underestimating overall RF exposure (in the worst‐case scenario). The second limitation was that the 5G measurements reported here only assessed one carrier per MNO, as the network optimization app only reported information on a single carrier and there was no control over which carrier the phone would use during measurements. Measurements across the spectrum, would however, include all carriers at any given site. Finally, as mentioned above, it was not always possible to trigger a maximum load in the direction of the measurement location.

## 5 Conclusions

The level of RF exposure at 30 publicly accessible locations in the UK close to active 5G base stations was found to be well within the 1998 ICNIRP public reference levels, and similar to what has been reported elsewhere. No statistically significant difference in 5G exposure levels was observed between beamforming and non‐beamforming sites. Exposure levels close to base stations were not found to be dependent on type of urban and suburban microenvironment. Although RF exposure was generally found to fall with distance, distance on its own was a poor predictor of exposure due to the various site‐dependent factors that affect exposure levels. Contribution from 5G to total RF exposure was found to be low, with 4G DL being the dominant source of RF across most sites. The level of 5G EMF exposure during streaming tests was found to be similar to background levels, which was attributed to the efficiency of the network, and suggests that background measurements are a good representation of typical exposure at current urban and suburban 5G sites. Extrapolation to maximum exposure, using the methodology from IEC 62232 (2025), showed that even in the unlikely case that most of the channel resources are used by a single user, exposure is still well within the 1998 ICNIRP public reference levels.

In summary, the results from this study support UKHSA advice that RF exposure measurements in the UK at publicly accessible locations near to base stations are consistently well within the ICNIRP guideline levels.

## Ethics Statement

The authors have nothing to report.

## Consent

The authors have nothing to report.

## Conflicts of Interest

The authors declare no conflicts of interest.

## Supporting information

Supplement 1.

Supplement 2.

## Data Availability

Data will be made available upon request.
