# Supplementary material for: In‐Situ Measurements of Radiofrequency Electromagnetic Fields Measurements Around 5G Macro Base Stations in the UK
Source: Bioelectromagnetics. 2025 Jun 30;46(5):e70012. doi: 10.1002/bem.70012 (PMC12207951; doi:10.1002/bem.70012)
Supplement: Supplementary file 1 — Supplement 1. [file BEM-46-0-s002.docx]

Supplement 1: Frequency bands used for the integration of environmental measurements.

| **Technology** | **F_min_ [MHz]** | **F_max_ [MHz]** | **Technology** | **F_min_ [MHz]** | **F_max_ [MHz]** |
| --- | --- | --- | --- | --- | --- |
| Broadcast TV | 470 | 700 | LTE UL | 1741.7 | 1781.7 |
| LTE UL | 700 | 713 | LTE/NR UL | 1781.7 | 1785 |
| NR UL | 723 | 733 | LTE DL | 1805.1 | 1831.7 |
| NR DL | 738 | 768 | GSM DL | 1831.7 | 1836.7 |
| LTE DL | 768 | 778 | LTE DL | 1836.7 | 1876.7 |
| NR DL | 778 | 788 | LTE/NR UL | 1876.7 | 1880 |
| LTE DL | 791 | 821 | DECT | 1880 | 1899.9 |
| LTE UL | 832 | 862 | LTE TDD | 1899.9 | 1909.9 |
| Zigbee | 863 | 876 | UMTS TDD | 1909.9 | 1920 |
| GSM UL | 876 | 879.9 | UMTS UL | 1920.3 | 1924.9 |
| UMTS UL | 880.1 | 890.1 | LTE/NR UL (DSS) | 1924.9 | 1964.7 |
| GSM UL | 890.1 | 892.5 | UMTS UL | 1959.7 | 1979.7 |
| GSM/UMTS/LTE UL | 892.5 | 902.5 | UMTS DL | 2110.3 | 2114.9 |
| UMTS/LTE UL | 902.5 | 912.5 | LTE/NR DL (DSS) | 2114.9 | 2149.7 |
| GSM UL | 912.5 | 914.9 | UMTS DL | 2149.7 | 2154.7 |
| GSM DL | 921 | 924.9 | LTE/NR DL (DSS) | 2154.7 | 2169.7 |
| UMTS UL | 925.1 | 935.1 | LTE TDD | 2340 | 2390 |
| GSM DL | 935.1 | 937.5 | WLAN | 2400 | 2483.5 |
| GSM/UMTS/LTE DL | 937.5 | 947.5 | ISM | 2483.5 | 2499 |
| UMTS/LTE DL | 947.5 | 957.5 | LTE UL | 2500 | 2570 |
| GSM DL | 957.5 | 959.9 | LTE TDD | 2575 | 2615 |
| Space Research | 960.5 | 1350 | LTE DL | 2620 | 2690 |
| LTE DL | 1452 | 1492 | NR TDD | 3300 | 4200 |
| LTE UL | 1710.1 | 1736.7 | WLAN | 5150 | 5850 |
| GSM UL | 1736.7 | 1741.7 |  |  |  |
