# Supplementary material for: In‐Situ Measurements of Radiofrequency Electromagnetic Fields Measurements Around 5G Macro Base Stations in the UK
Source: Bioelectromagnetics. 2025 Jun 30;46(5):e70012. doi: 10.1002/bem.70012 (PMC12207951; doi:10.1002/bem.70012)
Supplement: Supplementary file 2 — Supplement 2. [file BEM-46-0-s001.docx]

| **Country** | **Exposure levels** | **Distance** | **Line of sight (LOS)** | **Measurement type** | **Sample size, N** | **Measurement period** | **Reference** |
| --- | --- | --- | --- | --- | --- | --- | --- |
| Belgium, The Netherlands,  Germany, Switzerland | Maximum download traffic:  18.25 mWm^-2^ (0.66 – 63.69) mWm^-2^ | (62 - 300) m | Not specified | Measurement over the 5G band (3500 MHz) | Belgium: N=2,  Netherlands: N=2  Germany: N=1,  Switzerland: N=1 | 2021 | Deprez et al. 2022 |
| France | **Broadband (idle*):**  Avg = 3.76 mWm^-2^  SD = 1.66 mWm^-2^  Max = 90.16 mWm^-2^  **Channel power measurements:**  Idle*:  Avg = 0.07 mWm^-2^  SD = 0.07 mWm^-2^  Max = 3.95 mWm^-2^  Download of one single 1 GB file (6 min average):  Avg = 1.30 mWm^-2^  SD = 1.66 mWm^-2^  Max = 87.70 mWm^-2^ | Approx 100 m | Yes | - Broadband measurements - Measurement over the 5G band (3500 MHz) | Broadband measurements: N=1358  Channel power measurements: N = 464 | 2021 (after 8 months of 5G deployment at given site) | Sefsouf et al. 2024 |
| Greece | **All sites (idle*):**  Broadband: 46.79 mWm^-2^, (0.05 – 459.38) mWm^-2^  **Measurements at ground level close to a rooftop 5G base station, idle* (N=1):**  Broadband: 4.21 mWm^-2^,  5G band: 0.14 mWm^-2^ | Not reported | Not specified | - For all sites:   Broadband (420 MHz – 6 GHz)   - For single 5G base station:   Broadband and measurement over the 5G band (3400–3800 MHz) | N = 117 (for broadband measurements),  N=1 for measurements close to 5G BS. | 2021 until the mid of 2022 | Christopoulou et al. 2024 |
| Japan | Idle*:  Median values ranged between  (1 – 3) µW/m^2^ across 8 prefectures | Not applicable (drive measurements) | Not specified | Car-mounted band measurements over the 5G band | 530 | May 2021 and February 2022 | Onishi et al. 2023 |
| South Korea | **PEM EME SPY 200 data (range across locations):**  **Across all locations except close to 5G BS:**  Broadband: (0.04 – 8.03) mWm^-2^  5G band (average idle*): (<0.01 – 0.05) mWm^-2^  5G band (maximum idle*): (0.02 – 31.39) mWm^-2^  **Close to a 5G BS (distance not specified):**  Broadband: 0.24 mWm^-2^  5G band (avg idle*): 0.08 mWm^-2^  5G band (max idle*): 44.37 mWm^-2^  **Channel power data (BW=20 MHz) and with maximum traffic as set by operator:**  Street level (150 m from 5G BS) = 0.22 mWm^-2^  Rooftop (15 m from 5G BS) = 0.0006 mWm^-2^ | Distance provided for channel power measurements | PEM data: Not applicable  Channel power measurement: LOS | - PEM: Broadband (80 MHz – 6 GHz) and over 5G band (average and maximum from measurements at any given location) - channel power measurements | PEM: undefined  Channel power:  N = 1 | November 2019 | Selmaoui et al. 2021 |
| Switzerland | Idle*: (0.3 – 4.2) µWm^-2^  Maximum load: (1.1 – 424.4) µWm^-2^  Extrapolation: (26.5 – 954.9) µWm^-2^ | (30 – 410) m | LOS | Measurement over the 5G band (3500 MHz) and SSB zero span measurements | Four 5G base stations, 22 positions | July 2020 | Aerts, S et al. 2021 |

*idle: This means 5G downlink traffic was not generated during measurements.
